# Nitrogen-Rich Multinuclear Ferrocenophanes as Multichannel Chemosensor Molecules for Transition and Heavy-Metal Cations

**DOI:** 10.3390/s140814339

**Published:** 2014-08-07

**Authors:** Antonia Sola, Arturo Espinosa, Alberto Tárraga, Pedro Molina

**Affiliations:** Departamento de Química Orgánica, Facultad de Química, Universidad de Murcia, Campus de Espinardo, 30100 Murcia, Spain; E-Mails: antoniasola@um.es (A.S.); artuesp@um.es (A.E.)

**Keywords:** ferrocenophane, electrochemical sensing, chromogenic sensing, aldimine binding site, Zn(II) metal cations, Pb(II) metal cations, Hg(II) metal cations

## Abstract

[m.n] Multinuclear ferrocenophanes prepared by aza-Wittig reaction of bisiminophosphoranes derived from 1,1′-diazidoferrocene and isophthaladelhyde or 2,5-diformylthiophene, behave as efficient electrochemical and chromogenic chemosensor molecules for Zn^2+^, Pb^2+^, and Hg^2+^ metal cations. Whereas the OSWV of receptor **3**, bearing two *m*-phenylene units in the bridges, display one oxidation peak, receptor **4** incorporating two thiophene rings in the bridges, exhibits two well-separated oxidation peaks. In both receptors only the addition of Zn^2+^, Pb^2+^, and Hg^2+^ metal cations induced a remarkable anodic shift of ferrocene/ferrocenium redox couple. Likewise, in the absorption spectra of these receptors the low energy band is red-shifted by Δλ = 165 − 209 nm, and these changes promoted a significant color changes which could be used for the naked eye detection of these metal cations. The coordination modes for two representative cases were unveiled by DFT calculations that show an unsual coordination in the [**4**_2_Pb]^2+^ complex with the Pb^2+^ cation in a distorted cubic N_4_S_4_ donor cage.

## 1. Introduction

The development of simple and sensitive metal cation sensors continues to be a research area of considerable interest because of the important roles that these species play in biological, pathological or environmental processes. Among the various heavy metal cations, mercury and lead are especially hazardous because they are not biodegradable and can accumulate in the environment, which results in contaminated food and water, thus causing a variety of serious diseases such as neurological, metabolic, cognitive, kidney and motor disorders [1–6]. Consequently, the World Health Organization (WHO) and Environmental Protection Agency (EPA) have strictly defined their concentration limits in drinking water [7]. On the other hand, zinc is the second most abundant transition metal following iron and it plays well known roles in biological processes, the most important being as a structural cofactor in metalloproteins [8]. Thus, zinc metabolism disorders are closely associated with many severe neurological diseases such as Alzheimer's disease, amyotrophic lateral sclerosis and Parkinson's disease [9]. Therefore, during the last decades numerous efforts have been devoted to the development of abiotic receptors able to bind selectively cationic species with a concomitant change in one or more properties of the system, such as redox potentials, absorption or fluorescence spectra. In this context, the redox-active organometallic ferrocene scaffold has largely proved to be a simple and remarkably robust building block for the preparation of derivatives which have been considered as prototype chemosensor molecules displaying interesting electrochemical-sensing properties [10–16].

The advantage associated with the use of these functionalized ferrocene-containing ligands lies in the fact that, upon complexation with metal cations, they undergo significant perturbations of the ferrocene/ferrocinium redox couple and the values of the corresponding oxidation potential shifts are informative about the strength of the recognition event: the closer the binding site to the ferrocene unit, the higher oxidation potential shift. Despite the rich chemistry of ferrocene, [m.m]ferrocenophanes bridged by nitrogen-containing chains remain almost unexplored, and only the preparation and properties of some multinuclear nitrogen-rich [2.2]-, [3.3]-, and [4.4]ferrocenophanes have been reported [17–21].

In connection with our previous studies on the synthesis, structural characterization and properties of new families of azaferrocenophane ligands which incorporate binding sites in the bridge for the purpose of selective recognition and sensing of metal ions, we report herein the synthesis and sensing properties of two new highly preorganized tetraaza[7.7]ferrocenophane systems in which the two ferrocene units are linked to a thiophene or a benzene ring through two aldimine functions giving rise to the corresponding ferrocenophane framework. An interesting feature of the synthetic methodology used is that the starting 1,1′-*bis*(azido)ferrocene (**1**), has proved to be an excellent platform on which to build diazaferrocenophane frameworks. The combined effect of the binding capability of the aldimine moieties and the close proximity to the redox center makes this structural motif a likely candidate for displaying selective redox cation-sensing properties.

## 2. Experimental Section

### 2.1. General Information

Commercial starting materials were purchased to Aldrich (Madrid, Spain) and they were used without further purification. 1,1′-*Bis*(azido)ferrocene was prepared as described previously in the literature [17]. All reactions were carried out under N_2_ and using solvents which were dried by routine procedures. Melting points were determined on a Kofler hot-plate melting point apparatus and are uncorrected. ^1^H-NMR spectra were recorded on a Bruker AC 300 and 400. The following abbreviations for stating the multiplicity of the signals have been used; s (singlet), bs (broad singlet), d (doublet). Chemical shifts refer to signals of tetramethylsilane. High resolution electrospray (ESI) mass spectra were recorded on a Fisons AUTOSPEC 500 VG spectrometer. Cyclic Voltammetry (CV) and Osteryoung Square Wave Voltammetry (OSWV) techniques were performed with a conventional three-electrode configuration consisting of a carbon working and platinum auxiliary electrodes and a Ag/AgCl reference electrode. The experiments were carried out with a 10^−4^ M solution of sample in CH_2_Cl_2_ containing 0.1 M (*n*-C_4_H_9_)_4_NPF_6_ (TBAHP) as supporting electrolyte. All the potential values reported are relative to the decamethylferrocene (DMFc) couple at room temperature. Deoxygenation of the solutions was achieved by bubbling nitrogen for at least 10 min and the working electrode was cleaned after each run. The cyclic voltammograms were recorded with a scan rate of 0.1 Vs^−1^, while the OSWV were recorded at a scan rate of 100 mVs^−1^ with a pulse hight of 10 mV and a step time of 50 ms. Typically, receptor (10^−4^ M) was dissolved in the appropriate solvent (5 mL) and TBAHP (base electrolyte) (0.194 g) added. The guest under investigation was then added as a 2.5 × 10^−2^ M solution in CH_3_CN using a microsyringe whilst the electrochemical properties of the solution were monitored. DMFc was used as an external reference both for potential calibration and for reversibility criteria. UV-vis and emission spectra were recorded in the solvents and at the concentrations stated in the text and in the corresponding figure captions.

### 2.2. General Procedure for the Preparation of [7.7]ferrocenophanes **3**, **4** and **5**

To a solution of 1,1′-*bis*(azido)ferrocene (**1**, 0.1 g, 0.37 mmol) in dry THF (30 mL), Bu_3_P (0.31 mL, 1.2 mmol) was added. The resulting solution was stirred at room temperature and under nitrogen for 1.5 h. Then, the appropriate dialdehyde (0.37 mmol) was added and the reaction mixtures were refluxed for 12 h. On cooling, the resulting crude was crystallized from CH_2_Cl_2_/THF (1/5) to give the corresponding ferrocenophane.

#### 2.2.1. Bis[1,3-phenylene-bis(methylimino)][7.7]ferrocenophane (**3**)

Red solid, 0.05 g, 21%; m.p. > 300 °C. ^1^H-NMR (400 MHz, CD_2_Cl_2_): δ 8.05 (s, 4H, = C–H), 7.56 (s, 2H, Ar–H_2_), 7.40 (d, 2H, *J* = 7.6 Hz, Ar–H_5_), 6.98 (m, 4H, Ar–H_4_), 4.62 (bs, 8H, H_α_–Fc), 4.34 (bs, 8H, H_β_–Fc). HR-ESIMS *m/z*: calcd (C_36_H_28_N_4_Fe_2_, [M^+^ + 2]): 629.1080; found: 629.1092.

#### 2.2.2. Bis[thiophene-2,5-diylbis(methylimino)][7.7]ferrocenophane (**4**)

Purple solid, 0.085 g, 38%; m.p. > 300 °C. ^1^H-NMR (300 MHz, CD_2_Cl_2_): δ 8.33 (s, 4H, = C–H), 6.98 (s, 4H, thiophene), 4.74 (bs, 8H, H_α_–Fc), 4.28 (bs, 8H, H_β_–Fc). HR-ESIMS *m/z*: calcd (C_32_H_24_N_4_Fe_2_ S_2_, [M^+^ + 2]): 641.0218; found: 641.0221.

#### 2.2.3. Bis[1,10-phenanthroline-2,9-diylbis(methylimino)][7.7]ferrocenophane (**5**)

Pink solid, 0.110 g, 36%; m.p. > 300 °C. ^1^H-NMR (400 MHz, CD_2_Cl_2_): δ 8.63 (s, 4H, = C–H), 7.85 (d, 4H, *J* = 8.4 Hz), 7.70 (d, 4H, *J* = 8.4 Hz), 7.38 (s, 4H), 4.92 (bs, 4H), 4.82 (bs, 4H), 4.55 (bs, 4H), 4.43 (bs, 4H); HR-ESIMS *m/z*: calcd (C_48_H_32_N_8_Fe_2_, [M^+^ + 2]): 833.1536; found: 833.1529.

### 2.3. Computational Details

Quantum chemical calculations were performed with the ORCA electronic structure program package [22]. All geometry optimizations were run with tight convergence criteria using the B3LYP [23,24] functional together with the RIJCOSX algorithm [25] and the Ahlrichs' segmented def2-TZVP basis set [26,27], starting from preoptimized geometries obtained with the smaller def2-SVP basis set [28]. For Pb atoms the [SD(60,MDF)] effective core potential was used [29,30]. In all optimizations and energy evaluations, the latest Grimme's semiempirical atom-pair-wise correction (DFT-D3 methods), accounting for the major part of the contribution of dispersion forces to the energy, was included [31]. From these geometries obtained at the above mentioned level, all reported electronic data were obtained by means of single-point (SP) calculations using the same functional as well as the more polarized def2-TZVPP basis set. Reported energies are uncorrected for the zero-point vibrational term. The topological analysis of the electronic charge density, ρ(**r**), within Bader's Atoms-In-Molecules (AIM) methodology [32–34] was conducted using the AIM2000 software [35,36] and the wavefunctions (electron densities) generated with the Gaussian09 software package, [37] that were also used to perform the natural bond orbital (NBO) population analysis [38,39] with which Wiberg Bond Indices (WBI) [40] were obtained. Figures 6 and 7 were drawn with VMD [41].

## 3. Results and Discussion

### 3.1. Synthesis and Characterization of **3**, **4** and **5**

**Scheme 1.**
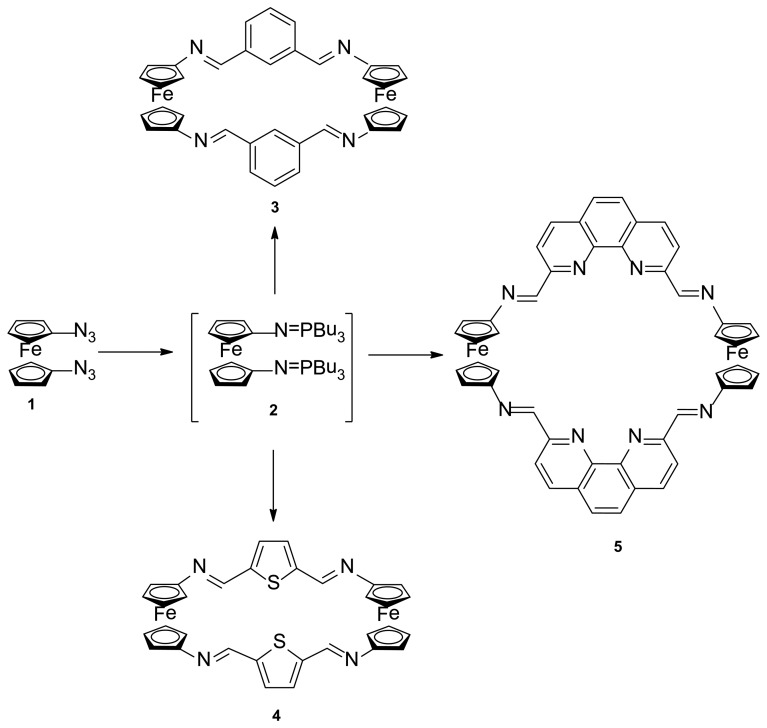
Synthetic route to [7.7]ferrocenophanes **3**, **4** and **5**.

The synthesis of the targets ferrocenophane derivatives **3**, **4** and **5** was carried out as depicted in Scheme 1, through a two-step procedure, starting from the previously reported 1,1′-*bis*(azido)ferrocene (**1**) [17], which in turn is available by the sequential treatment of ferrocene with *n*-BuLi followed by reaction with the strong azide-transfer agent 2,4,6-triisopropylbenzenesulfonyl azide (trisyl azide). Thus, compound **1** underwent Staudinger reaction with *n*-tributylphosphine under anhydrous conditions to give the not isolable *bis*-iminophosphorane **2** which, subsequently, underwent an aza-Wittig reaction with the appropriate dialdehyde in dry THF giving rise to the aforementioned ferrocenophanes **3**, **4** and **5** in 21%, 38% and 36% yield, respectively.

The structure of these compounds was elucidated using spectra ^1^H-NMR measurements as well as high resolution electrospray mass spectra (HR ESI-MS)]. In general, all the ^1^H NMR spectra of showed the presence of two pseudotriplets, integrating eight protons each, assigned to the Hα and Hβ protons within the symmetrically 1,1′-disubstituted ciclopentadienyl (Cp) rings present in the two equivalent ferrocene units. Additionally, one singlet, corresponding to the four iminic protons present in the bridges, together with the pattern of signals corresponding to the linked benzene, thiophene or 1,10-phenanthroline fragment was also observed (see ESI).

*Reagents and conditions*: (a) *n*-Bu_3_P, dry THF, 1.5 h, rt, N_2_; (b) isophthalaldehyde, dry THF, reflux 12 h; (c) 2,5-diformylthiophene, dry THF, reflux 12 h; (d) 2,9-diformyl-1,10-phenanthroline, dry THF, reflux 12 h.

### 3.2. Metal Cation Sensing Studies

An interesting common attribute of the new ferrocenophanes reported here is the presence of N-donor atoms on the bridges properly arranged with respect to the ferrocene redox-active moieties. Consequently, the receptors **3**, **4** and **5** are good candidates to study their coordination behavior towards several metal cations, not only through electrochemical methods, [linear sweep voltammetry (LSV), cyclic voltammetry (CV), and Osteryoung square-wave voltammetry (OSWV)] [42] but also by UV-vis and ^1^H-NMR spectroscopic techniques, and quantum chemical calculations. However, it should be mentioned that although 1,10-phenanthroline is a classic chelating bidentate ligand whose nitrogen atoms are placed to act cooperatively in cation binding [43], the low solubility of receptor **5** in the common organic solvents, promoted that the recognition studies were only focused on the ferrocenophanes **3** and **4**. We have found that all the iminoferrocenophane derivatives described in this paper are stable enough to undergo all the titration experiments without suffering any degradation. Only in the presence of traces of mineral acids does hydrolysis take place in a short period of time. To gain insight into the binding properties of both ligands, a set of alkali-metal ions (Li^+^, Na^+^ and K^+^), alkaline-earth metal ions (Mg^2+^ and Ca^2+^) and transition-metal metal ions (Ni^2+^, Cu^2+^, Zn^2+^, Cd^2+^, Hg^2+^, and Pb^2+^) as their triflate or perchlorate salts, were tested [44]. The titration experiments were further analyzed using the computer program SPECFIT [45].

#### 3.2.1. Electrochemical Study

The reversibility and relative oxidation potential of the ferrocene/ferrocenium redox couple in receptors **3** and **4** were determined by cyclic voltammetry (CV) and Osteryoung square-wave voltammetry (OSWV) in solutions of CH_2_Cl_2_ (c = 1 × 10^−4^ M) containing 0.1 M [(*n*-Bu)_4_N]PF_6_ as supporting electrolyte.

**Figure 1.**
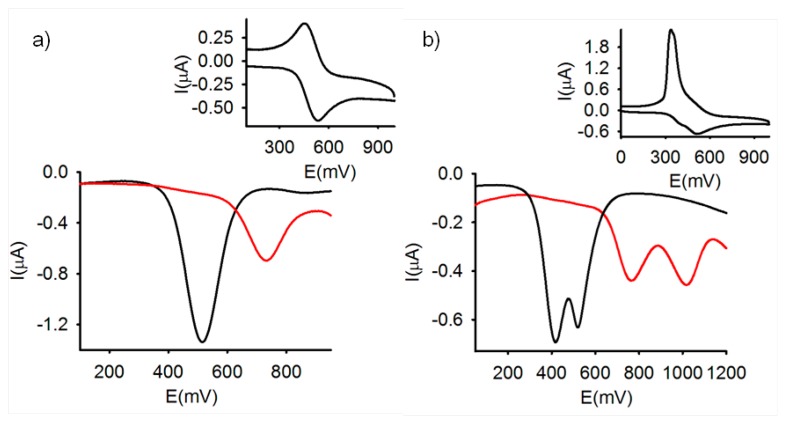
Evolution of the OSWV for the receptors **3** (**a**) and **4** (**b**) in CH_2_Cl_2_ (c = 1 × 10^−4^ M) (black line) upon addition of 1.4 equiv of Pb^2+^ (2.5·× 10^−2^ M en CH_3_CN) (red line), using [*n*-Bu_4_N]PF_6_ as supporting electrolyte. In inset, the corresponding CVs for both receptors are shown.

As expected, the CV of the free receptor **3** in CH_2_Cl_2_ (c = 1 × 10^−4^ M), showed a reversible two-electron oxidation wave at *E_1/2_* = 517 mV *vs* the decamethylferrocene (DMFc) redox couple indicating that the two metal centers are electronically decoupled. Similarly, OSWV also exhibits one oxidation peak at the same potential *vs* DMFc (Figure 1). By contrast the ferrocenophane **4** bearing the two redox centers separated by two 2,5-iminomethyl disubstituted thiophene bridges gave rise, under similar electrochemical conditions, to two successive one-electron oxidations at *E^1^_1/2_* = 420 and *E^2^_1/2_* = 530 mV, respectively (Figure 1), indicating the existence of an electronic interaction between the two organoiron centers, through such organic bridge.

It is worth mentioning that optically induced intramolecular electron transfer processes have been previously reported in electrochemically active ferrocenyl-thiophene derivatives [46–50]. Moreover, it has also been reported that the intramolecular electron transfer phenomena can be monitored by the study of the intervalence charge-transfer bands (IVCT) occurring in the electrochemically oxidized state of this type of π-bridged systems [51–55]. However, the detection of this IVCT bands through spectroelectrochemical experiments followed by UV-vis-near IR spectrocopy was unsuccessful due to the insolubility of this compound in the common organic solvents. Nevertheless, it has also been shown that the magnitude of the separation ΔE_1/2_ between the first and second redox events gives an indication of the interaction through the bridge between the two Fe sites [56]. In the present work this value has been calculated by the Richardson-Taube method [57] (E^1^_1/2_ = 420 mV, E^2^_1/2_ = 530 mV, cf ferrocene 570 mV *vs*. DMFc). From the separation |E^2^_1/2_ − E^1^_1/2_|= 110 mV a comproportionation constant Kc = 74 was calculated, which indicates that the monocation [**4**]^+^ could be an example of a slightly delocalized mixed-valence species.

The electrochemical changes upon addition of the above mentioned set of metal cations to the receptors **3** and **4** were also investigated by using OSWV. In the case of **3**, these experiments demonstrated that only the addition of Zn^2+^, Hg^2+^ and Pb^2+^ induced the appearance, in the OSWV, of a new oxidation peak at a remarkably more positive potential: E_1/2_ = 635 mV (ΔE_1/2_ = 118 mV) for Zn^2+^, E_1/2_ = 729 mV (ΔE_1/2_ = 212 mV) for Pb^2+^, and E_1/2_ = 745 mV (ΔE_1/2_ = 228 mV) for Hg^2+^ (Figures 1a, S1 and S2). Interestingly, while addition Zn^2+^, Pb^2+^ and Hg^2+^ metal cations to **3**, promotes the formation of the corresponding complexes, addition of Cu^2+^ induces the oxidation of the ferrocene moieties present in the free receptor. Thus, LSV studies carried out upon addition of Cu^2+^ to the CH_2_Cl_2_ electrochemical solutions of receptor **3**, showed a significant shift of the sigmoidal voltammetric wave toward cathodic currents, indicating that this metal cation promotes the oxidation of the free receptors. By contrast, the same experiments carried out upon addition of Zn^2+^, Hg^2+^ and Pb^2+^ metal cations, revealed a shift of the linear sweep voltammogram toward more positive potentials, which is in agreement with the complexation process previously observed by CV and OSWV (Figure S3).

On the other hand, the results obtained on the stepwise addition of substoichiometric amounts of the metal cations to receptor **4**, under the same electrochemical conditions, also revealed that only the addition of Zn^2+^, Hg^2+^ and Pb^2+^ metal cations promoted the appearance of two new waves anodically shifted (E^1^_1/2_ = 640 mV and E^2^_1/2_ = 954 mV for Zn^2+^, E^1^_1/2_ = 780 mV and E^2^_1/2_ = 1040 mV for Pb^2+^, and E^1^_1/2_ = 780 mV and E^2^_1/2_ = 805 mV for Hg^2+^) while the addition of the other metal cations tested had no effect on its CV or OSWV even when they were added in a large excess (Figures 1b and S4).

#### 3.2.2. UV-vis Absorption Study

**Figure 2.**
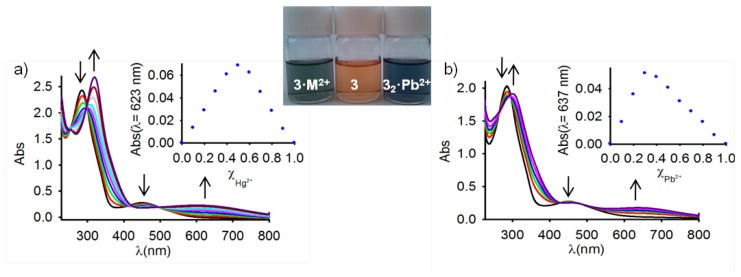
Evolution of the Uv-vis spectrum of **3** (c = 5·× 10^−5^ M, in CH_2_Cl_2_) upon addition of increasing amounts of (**a**) Hg^2+^, and (**b**) Pb^2+^ (c = 2.5·× 10^−2^ M in CH_3_CN) until 2 equiv. The arrows indicate the absorptions that increase or decrease during the experiment. Inset: Job's plots showing the 1:2 and 1:1 (M^+2^/receptor) stoichiometries for the complexes formed with Pb^2+^ and Hg^2+^, respectively.

**Figure 3.**
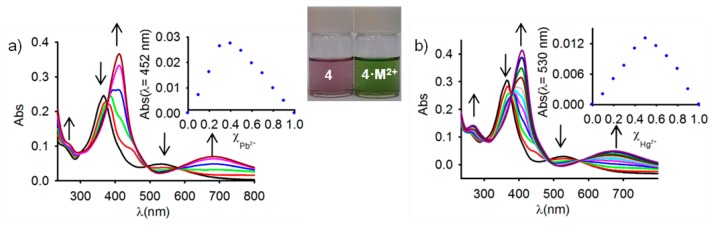
Evolution of the Uv-vis spectrum of **4** (c = 1·× 10^−4^ M, in CH_2_Cl_2_) upon addition of increasing amounts of (**a**) Pb^2+^, and (**b**) Hg^2+^ (c = 2.5·× 10^−2^ M in CH_3_CN) until 2 equiv. The arrows indicate the absorptions that increase or decrease during the experiment. Inset: Job's plots showing the 1:2 and 1:1 (M^+2^/receptor) stoichiometries for the complexes formed with Pb^2+^ and Hg^2+^, respectively.

Because studies on ferrocene-based ligands have clearly shown that their characteristic low-energy bands in the absorption spectra are perturbed on complexation [58–60], the cation binding properties of the ferrocenophanes **3** and **4** were also examined using UV-vis spectroscopic measurements. The absorption properties of both free receptors were first determined in CH_2_Cl_2_ and are summarized in Table 1. In both cases, a characteristic absorption band, present in most of the ferrocenyl derivatives, is detected at λ = 450 and λ = 532 nm. This weak absorption is produced either by two nearly degenerate transitions: a Fe(II) d-d transition [61,62] or by a metal-ligand charge transfer (MLCT) process (dπ − π*). This assignment is in agreement with the latest theoretical treatment (model III) reported by Barlow *et al.* [63]. The optical detection capability of these receptors toward the above mentioned set of metal cations was carried out by the progressive addition of the corresponding metal cations into a solution of them in CH_2_Cl_2_. These titration experiments confirmed the electrochemical results previously shown in the sense that only the presence of Zn^2+^, Hg^2+^ and Pb^2+^ metal cations displayed modifications of the UV-vis spectrum of the free receptors, as a consequence of its coordination to those metal cations, while no significant spectral changes were observed upon addition of the other metal cations tested. The changes observed during such coordination processes are similar in both cases (Figures 2, 3, S5 and S7 and Table 1). In particular, the lower energy band in **3** shifts from λ = 450 nm to higher wavelengths (λ = 642 nm for Zn^2+^, λ = 615 nm for Hg^2+^, and λ = 659 nm for Pb^2+^), a similar trend being also observed for the case of receptor **4**, in which the lower energy band appearing at λ = 532 nm is also red shifted to λ = 675 nm, λ = 680 nm, and λ = 685 nm, upon addition of Zn^2+^, Hg^2+^, and Pb^2+^, respectively. These progressive changes in the spectra revealed clear isosbestic points, indicating the presence of only two species in equilibrium in the solution, namely, the free receptor and the corresponding metal complex. As shown in Figures 2 and 3, such changes also promoted a significant colour changes in the solutions of the free receptors which could be used for the naked eye detection of these metal cations. Furthermore, Job's plots and titration profiles obtained on the basis of the changes in the absorption spectra upon addition of these metal cations also indicated 1:1 stoichiometries for the complexes between both receptors and Zn^2+^ and Hg^2+^, and 1:2 stoichiometries for the complexes formed by Pb^2+^ metal cations. Moreover, the calculated association constant [45] and detection limits [64] (Figures S6 and S8) are collected in Table 1.

#### 3.2.3. ^1^H-NMR Study

**Figure 4.**
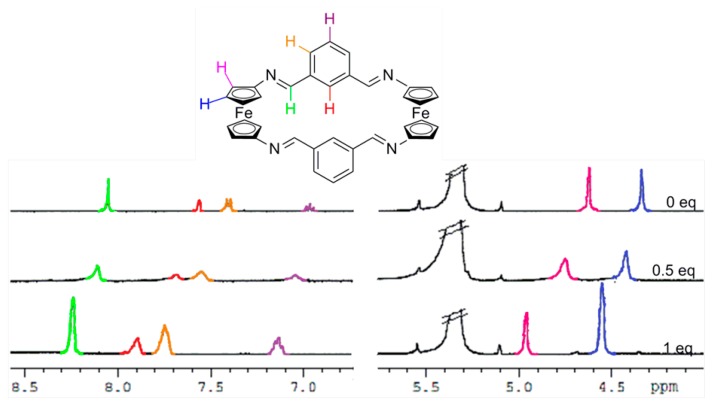
Evolution of the ^1^H-NMR spectrum of the free ligand **3**, in CD_2_Cl_2_, upon addition of increasing amounts of Zn^2+^ until 1 equiv. The crossed signal corresponds to the solvent.

**Figure 5.**
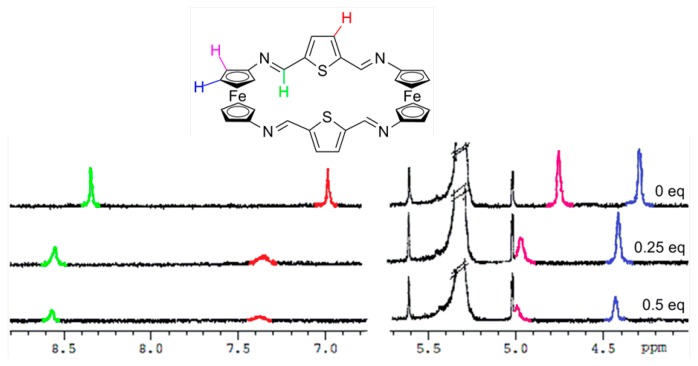
Evolution of the ^1^H-NMR spectrum of the free ligand **4**, in CD_2_Cl_2_, upon addition of increasing amounts of Pb^2+^ until 0.5 equiv. The crossed signal corresponds to the solvent.

In order to gain further understanding on the recognition processes, ^1^H-NMR titration experiments in CD_2_Cl_2_ were carried out. As shown in Figures 4, S9 and S10, and in Table 2, the most significant changes observed upon the gradual addition of Zn^2+^, Hg^2+^ and Pb^2+^ metal cations to receptor **3** are the clear downfield shifts promoted in the protons present within both the ferrocene and the bridge units. Similarly, the two single peaks assigned for the four iminic protons, the four thiophene protons as well as the eight Hα and eight Hβ protons within the 1,1'-disubstituted ferrocene units present in **4** were also downfield shifted (Figure 5, S11 and S12 and Table 2). However, it is worth mentioning that, in both receptors, the ferrocenyl protons were distinctly downfield shifted upon complexation, indicating the different electron deshielding effect promoted by the metal cations bound in the proximity of such ferrocene moieties. Moreover, the strong thiophilic affinity by Hg^2+^ is also clearly evidenced in receptor **4** where the downfield shifts promoted upon complexation are significantly higher than in the species [**3**·Hg^2+^]. Moreover, the observed downfield shifts of the thiophene protons in **4** upon recognition of Hg^2+^, in comparison to other metal cations, also indicates the cooperative role that the sulfur atom, present in this rigid structural motif, should play during the recognition process. In addition, the variation of ^1^H NMR spectra upon titration with Pb^2+^ show that both receptors **3** and **4** experienced saturation of the change in chemical shift when 0.5 equiv of Pb^2+^ was added, while in the cases of Zn^2+^, and Hg^2+^ such saturation was achieved when 1 equiv of these metal cations was added.

#### 3.2.4. Computational Study

Computational calculations were carried out to find out the binding mode of the Zn^2+^ and Hg^2+^ metal cations with receptor **4**. QC calculations show that the most stable geometry for ligand **4** (see the SI) features a parallel alignment of the two (*Z*,*Z*)-2,5-thienyl-bisiminoyl bridges at typical π-stacking distance (*ca.* 3.62 Å at thiophene α positions). Four main conformers of **4** were compared at a preliminary B3LYP-D3/def2-SVP level of theory. The most stable arc-shaped *Z*,*Z*-bridges allow the thiophene units to be far away (*i.e.*, no stacked) in the “antiparallel” arrangement **4***^ZZ^***^−anti^** that was found to be 3.73 kcal/mol less stable. This energy difference could be used as a rough estimation of the magnitude for the π-stacking interaction in **4** [65], that is additionally evidenced by one bond critical point (BCP) between both S atoms (d_S⋯S_ = 3.669 Å; WBI = 0.002; ρ(r) = 0.69 × 10^−2^ *e/a_o_^3^*), as well as two other BCPs between both formal thienyl diene moieties (average d_C⋯C_ = 3.609 Å; ΣWBI = 0.010; Σρ(r) = 0.91 × 10^−2^ *e/a_o_^3^*) and one BCP between each C = N pair (WBI = 0.008; ρ(r) = 0.41 × 10^−2^ *e/a_o_^3^*).

**Figure 6.**
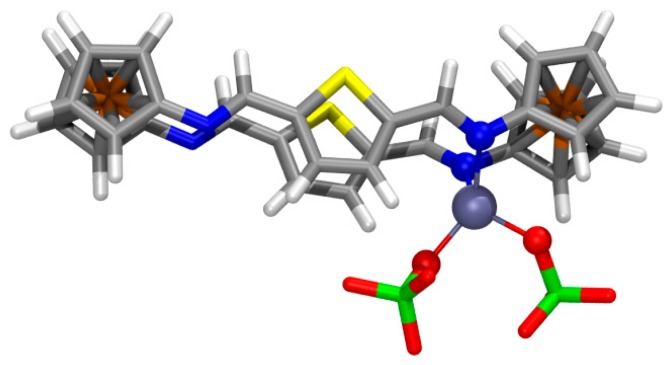
Computed (B3LYP-D3/def2-TZVP) most stable geometry for complex **4**·Zn(ClO_4_)_2_. Capped sticks representation except for the central metal cation and directly linked donor atoms that are highlighted in ball representation.

Among several different structural possibilities computed for the 1:1 complex of **4** with Zn(ClO_4_)_2_, the most stable geometry (Figure 6) displays the ligand with *E*,*E*-configured bridges and the metal cation chelated by two imine N atoms (d_Zn-N_ = 2.115, 2.143 Å; WBI = 0.186, 0.182; ρ(r) = 6.79 × 10^−2^, 6.43 × 10^−2^ *e/a_o_^3^*; angle N-Zn-N: 89.1°). The parallel alignment of the bridges is significantly distorted by elongating the S⋯S distance (d_S⋯S_ = 4.170 Å; ρ(r) = 0.29 × 10^−2^ *e/a_o_^3^*) thus allowing the approach of both chelating imine N atoms (d_N⋯N_ = 2.987 Å) [66]. The *E*-configuration at the imine groups allows the formation of two moderately strong hydrogen bonds (HB) of one perchlorate unit with thienyl H atoms (d_O⋯H_ = 2.009, 2.204 Å; WBI = 0.015, 0.012; ρ(r) = 2.42 × 10^−2^, 1.60 × 10^−2^ *e/a_o_^3^*), whereas two other weaker HB are formed between the other perchlorate unit and ferrocenyl H atoms (d_O⋯H_ = 2.184, 2.287 Å; WBI = 0.005, 0.009; ρ(r) = 1.68 × 10^−2^, 1.27 × 10^−2^ *e/a_o_^3^*).

**Figure 7:**
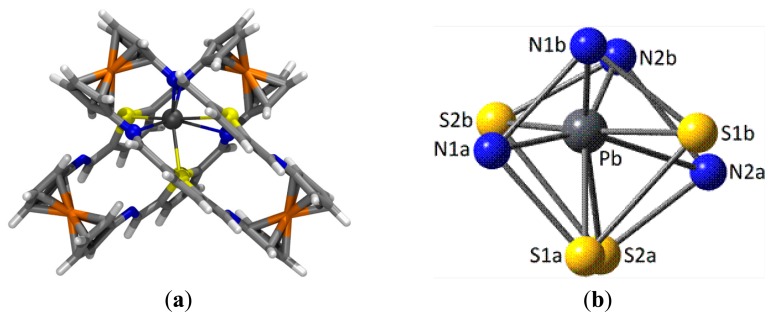
(**a**) Computed (B3LYP-D3/def2-TZVPecp) most stable geometry for complex **4**_2_·Pb^2+^. Capped sticks representation except for the central metal cation and directly linked donor atoms that are highlighted in ball representation; (**b**) Simplified coordination sphere around the central Pb^2+^ cation.

In the case of the complex with Pb^2+^ cations and taking into account the experimentally obtained 2:1 ligand to metal stoichiometry, the most stable geometry for the **4**_2_·Pb^2+^ complex (Figure 7a) shows an unusual distorted cubic 8-coordination around the metal cation, made up by four N and four S atoms. To some extent, the highly distorted coordination sphere arises from the fact that two ligands (labeled as “1” and “2”) in their most stable parallel *Z*,*Z*-arrangement **4**, must approach each other using a four donor atoms S_2_N_2_ set each, consisting of one S and one N atoms belonging to one bridge (labeled as “a”) and the corresponding atoms in the other bridge (“b”). As a result of the geometrical constraints in the ligands, all donor atoms approach leading to one S⋯S and one N⋯N (homo)pairings and two S⋯N (hetero)pairings (Figure 7b).

The central Pb^2+^ cation lies very much closer to the N1b⋯N2b (d_Pb-N1b_ = 2.611 Å, WBI = 0.126; ρ(r) = 4.19 × 10^−2^ *e/a_o_^3^*; d_Pb-N2b_ = 2.733 Å, WBI = 0.117; ρ(r) = 3.34 × 10^−2^ *e/a_o_^3^*) and the N1a⋯S2b pairs (d_Pb-N1a_ = 2.899 Å, WBI = 0.115; ρ(r) = 2.41 × 10^−2^ *e/a_o_^3^*; d_Pb-S2b_ = 3.082 Å, WBI = 0.161; ρ(r) = 2.52 × 10^−2^ *e/a_o_^3^*). The other three S donor atoms bind the metal with moderate strength (d_Pb-S1b_ = 3.113 Å, WBI = 0.153; ρ(r) = 2.33x10^−2^ *e/a_o_^3^*; d_Pb-S1a_ = 3.310 Å, WBI = 0.129; ρ(r) = 1.52 × 10^−2^ *e/a_o_^3^*; d_Pb-S2a_ = 3.417 Å, WBI = 0.124; ρ(r) = 1.27x10^−2^ *e/a_o_^3^*), whereas the interaction with the remaining N2a atom is remarkably long (d_Pb-N2a_ = 3.533 Å, WBI = 0.070; ρ(r) = 0.72 × 10^−2^ *e/a_o_^3^*). Very likely some long contacts (especially that with N2a) within the distorted cubic coordination is a consequence of the need for a *hemidirected* environment around the metal allowing some room to accommodate the stereochemical electron pair [67,68], characteristic of Pb(II) and other species. In addition, the structure of the **4**_2_·Pb^2+^ complex is further stabilized by secondary interactions of HB or T-stacking nature between the two ligand units.

## 4. Conclusions

The synthesis and electrochemical, optical, and ion sensing properties of [m.n]ferrocenophanes with bridges decorated with aldimines as cation-binding sites and an aromatic or heteroaromatic (thiophene) spacer, are presented. Both receptors act as efficient redox/chromogenic chemosensor molecules for Zn^2+^, Pb^2+^, and Hg^2+^ cations in CH_3_-CN, whereas Cu^2+^ cations induced oxidation of the ferrocenyl end groups, which is confirmed by linear sweep voltammetry (LSV) data. The absorption spectra changes are accompanied by a color change which suggests the potential for “naked eye” detection. It is worth mentioning that from the calculated Kas and detection limits no pronounced differences in terms of selectivity and sensitivity of the highly preorganized ligands **3** and **4** toward Zn^2+^, Pb^2+^, and Hg^2+^ cations were observed, probably due to the fact that the ferrocenophane hole is not involved in the coordination mode. DFT calculations reveal an unusual distorted cubic coordination for the Pb^2+^ cation in the [**4**_2_Pb]^2+^ complex in a N_4_S_4_ donor cage.

## Figures and Tables

**Table 1.** Characteristic UV-Vis data for the receptors **3** and **4** and their metal complexes.

**Comp.**	**UV-vis λ_max_ (10^−3^ ε) ^a^**	**IP ^b^**	**K_as_**	**D_lim_^c^**
**3**	285(20.27), 450(2.73)	-	-	-
**3·Zn^2+^**	312(23.03), 469(2.27), 642(2.23)	497, 419, 298, 245	1.44 × 10^6 e^	7.1 × 10^−6^
**3·Hg^2+^**	320(26.81), 615(2.26)	496, 419, 298, 251	4.58 × 10^5 e^	1.4 × 10^−5^
**3·Pb^2+^**	296(18.69), 469(2.53), 659(1.54)	484, 427, 296, 263	1.26 × 10^8 d^	6.7 × 10^−6^
**4**	250(5.06), 367(12.27), 532(2.41)	-	-	-
**4·Zn^2+^**	269(5.99), 392(17.71), 408(18.12), 675(1.97)	570, 489, 375	2.86 × 10^−6 e^	1.5 × 10^−6^
**4·Hg^2+^**	268(7.04), 408(20.68), 680(2.47)	574, 497, 377	2.17 × 10^6 e^	1.8 × 10^−5^
**4·Pb^2+^**	272(6.12), 411(21.00), 685(3.86)	588, 489, 376, 298	5.89 × 10^9 d^	6.7 × 10^−6^

a ε in·dm^3^·mol^−1^·cm^−1^;

b isosbestic points in nm;

c detection limits in M;

d in M^−2^;

e in M^−1^.

**Table 2.** Deshielding effect promoted in receptors **3** and **4** by the metal cations.

**Comp.**	**Δδ_CH=N_**	**Δδ_H2_**	**Δδ_H4_**	**Δδ_H5_**	**Δδ_CH thiop_**	**Δδ_Hα_**	**Δδ_Hβ_**
**3·Zn^2+^**	0.20	0.34	0.33	0.19	-	0.32	0.22
**3·Hg^2+^**	0.35	0.52	0.43	0.24	-	0.44	0.27
**3·Pb^2+^**	0.12	0.23	0.25	0.13	-	0.22	0.15
**4·Zn^2+^**	0.32	-	-	-	0.56	0.33	0.15
**4·Hg^2+^**	1.12	-	-	-	1.21	0.92	0.38
**4·Pb^2+^**	0.21	-	-	-	0.37	0.22	0.12
